# Vision-Related Quality of Life and Dry Eye Symptoms After Treatment with Lyophilized Amniotic Membrane Eye Drops: A Secondary Analysis of a Prospective Cohort Study

**DOI:** 10.3390/jcm15197568

**Published:** 2026-09-29

**Authors:** Jelena Kostic, Svetlana Stanojlovic, Borivoje Savic, Bojana Dacic Krnjaja, Tanja Kalezic, Vladimir Milutinovic, Nada Avram, Bozidar Savic, Nataša Maksimovic

**Affiliations:** 1 Clinic for Eye Diseases, University Clinical Center of Serbia, 11000 Belgrade, Serbia; 2 Faculty of Medicine, University of Belgrade, 11000 Belgrade, Serbia; 3 Faculty of Medicine Foča, University of East Sarajevo, 73300 Foča, Bosnia and Herzegovina; 4 Institute of Veterinary Medicine of Serbia, 11000 Belgrade, Serbia; 5 Institute of Epidemiology, Faculty of Medicine, University of Belgrade, 11000 Belgrade, Serbia

**Keywords:** dry eye disease, lyophilized amniotic membrane, amniotic membrane eye drops, patient-reported outcomes, vision-related quality of life, NEI VFQ-25, OSDI-6, tear film stability, ocular surface disease

## Abstract

**Background/Objectives**: Dry eye disease (DED) affects daily visual functioning and vision-related quality of life in addition to tear film stability and ocular surface integrity. In a previously published prospective cohort, lyophilized amniotic membrane (AM) eye drops were associated with significant improvements in objective ocular surface parameters. This secondary analysis evaluated whether treatment with lyophilized AM eye drops was accompanied by changes in patient-reported outcomes, specifically vision-related quality of life and dry eye symptoms. **Methods**: This secondary analysis included 40 consecutive patients with DED treated with lyophilized AM eye drops in repeated 14-day cycles over six study visits, while continuing their pre-existing standard DED therapy. Patient-reported outcomes were assessed at baseline and at the final visit using the National Eye Institute Visual Function Questionnaire-25 (NEI VFQ-25) and the Ocular Surface Disease Index-6 (OSDI-6). Item-level response distributions were compared between baseline and the final visit using non-parametric tests for paired ordinal data. A two-sided *p* value < 0.05 was considered statistically significant. **Results**: Global NEI VFQ-25 items, including general health, self-rated vision, and worry about eyesight, did not change significantly. In contrast, significant improvements were observed in ocular pain/discomfort (*p* = 0.002); near-vision activities, including reading ordinary print (*p* = 0.003), work or hobbies requiring near vision (*p* = 0.014); and finding objects on a crowded shelf or in a drawer (*p* = 0.008), as well as distance-related and spatial tasks, including reading street signs (*p* < 0.001) and noticing objects off to the side while walking (*p* = 0.037). Improvements were also observed in vision-related social functioning and dependence-related items. Five of six OSDI-6 items improved significantly: light sensitivity (*p* < 0.001), blurred vision (*p* = 0.008), difficulty with driving or night driving (*p* = 0.002), difficulty watching television (*p* = 0.008), and discomfort in windy conditions (*p* = 0.046). Discomfort in low-humidity environments showed a trend toward improvement but did not reach statistical significance (*p* = 0.059). **Conclusions**: In this secondary analysis of a prospective cohort, lyophilized AM eye drops were associated with significant improvements in several patient-reported domains of vision-related quality of life and dry eye symptoms. These findings complement previously reported objective improvements in tear film stability, ocular surface staining, meibomian gland function, and corneal sensitivity, suggesting that treatment with lyophilized AM eye drops was associated with improvements extending beyond ocular surface signs to patient-reported daily visual function and symptom burden in patients with DED.

## 1. Introduction

Dry eye disease (DED) is a chronic and multifactorial disorder of the ocular surface characterized by loss of tear film homeostasis, tear film instability, ocular surface damage, inflammation, and neurosensory abnormalities [1,2]. Although DED is traditionally evaluated using objective clinical parameters such as tear break-up time (TBUT), ocular surface staining, Schirmer testing, and meibomian gland assessment, it is also a highly symptomatic condition with a substantial impact on daily visual functioning and vision-related quality of life [3,4,5,6]. Patients frequently report ocular discomfort, burning, foreign-body sensation, photophobia, fluctuating or blurred vision, and visual fatigue, which may interfere with reading, computer use, driving, watching television, social interaction, and work productivity [5,6].

A central challenge in the clinical evaluation of DED is the well-recognized discordance between objective signs and subjective symptoms. Some patients report severe symptoms despite relatively mild ocular surface findings, whereas others show marked clinical abnormalities with limited symptom reporting [7,8]. For this reason, contemporary DED assessment increasingly emphasizes the combined evaluation of objective ocular surface parameters and patient-reported outcomes. Such an approach is consistent with the TFOS DEWS II framework, which recognizes that treatment success should not be defined solely by improvement in tear film stability or ocular surface staining, but also by the extent to which patients experience meaningful relief of symptoms and improvement in daily visual function [1,2,3,4].

Validated questionnaires play an important role in capturing this patient-centered dimension of DED. The National Eye Institute Visual Function Questionnaire-25 (NEI VFQ-25) is a widely used instrument for assessing vision-related quality of life across multiple domains, including general vision, ocular pain, near and distance activities, social functioning, mental health, role limitations, dependency, driving, color vision, and peripheral vision [9]. In contrast, the Ocular Surface Disease Index (OSDI) focuses more directly on the frequency and functional impact of dry eye symptoms [10]. The shortened six-item version, OSDI-6, was developed as a concise instrument for assessing key symptom and functional domains of ocular surface disease [11]. The combined use of NEI VFQ-25 and OSDI-6 may therefore provide complementary information: NEI VFQ-25 captures the broader functional and psychosocial consequences of visual impairment, whereas OSDI-6 provides a focused assessment of core dry eye symptom burden. Patient-reported outcome measures in DED differ in content, psychometric quality, and clinical applicability; therefore, careful selection and interpretation of questionnaires are essential when evaluating treatment response from the patient’s perspective [12].

Amniotic membrane (AM)-based therapies have gained increasing interest in ocular surface disease because of their extracellular matrix-rich structure, anti-inflammatory and pro-resolving properties, and potential epithelial and neurotrophic effects [13,14,15,16,17,18]. AM-derived eye drop preparations contain bioactive matrix components, growth factors, and immunomodulatory mediators that may support epithelial repair, reduce inflammatory activation, and improve ocular surface stability [14,15,16,17]. These biological properties are particularly relevant in DED, where tear film instability, epithelial barrier dysfunction, meibomian gland abnormalities, inflammation, and neurosensory dysfunction frequently coexist and jointly contribute to symptom burden and reduced quality of life [1,2].

Most available clinical studies of AM-derived eye drops have primarily focused on objective ocular surface outcomes, including TBUT, ocular surface staining, epithelial healing, meibomian gland parameters, and safety [14,15,16,17]. In addition, clinical studies of other AM-based ocular surface therapies have also reported improvement in signs and symptoms of DED [18]. However, the extent to which these objective improvements translate into patient-perceived benefit remains insufficiently characterized. In particular, data on vision-related quality of life and structured dry eye symptom outcomes after treatment with AM-derived eye drops remain limited. This represents an important evidence gap, because a therapy that improves ocular surface signs without improving symptoms or daily visual function may have limited perceived value from the patient’s perspective.

In a previously published prospective cohort study, lyophilized AM eye drops were associated with significant improvements in objective clinical signs of DED, including prolongation of TBUT, reduction in corneal and conjunctival staining, improvement in meibomian gland quality and expressibility, and increased corneal sensitivity, while best-corrected visual acuity, intraocular pressure, and Schirmer I values remained stable [19]. The observed pattern suggested that lyophilized AM eye drops primarily improved tear film quality and ocular surface integrity rather than aqueous tear production.

The present study represents a secondary analysis of the same prospective cohort, focusing specifically on patient-reported outcomes. The aim of this study was to evaluate changes in vision-related quality of life and dry eye symptoms, assessed using the NEI VFQ-25 and OSDI-6 questionnaires, in patients with DED treated with lyophilized AM eye drops. In addition, we aimed to interpret these patient-reported changes in relation to the previously observed objective improvements in tear film stability and ocular surface parameters, in order to determine whether clinical stabilization of the ocular surface was accompanied by meaningful improvement in patients’ daily visual function and symptom burden.

## 2. Materials and Methods

### 2.1. Study Design and Ethical Approval

This study was designed as a secondary analysis of a previously published prospective cohort study evaluating the clinical effects of lyophilized AM eye drops in patients with DED [19]. The primary publication focused on objective ocular surface outcomes, including TBUT, corneal and conjunctival staining, meibomian gland parameters, corneal sensitivity, Schirmer I test, best-corrected visual acuity, and intraocular pressure. The present analysis focuses specifically on patient-reported outcomes, namely vision-related quality of life and dry eye symptoms, assessed using the NEI VFQ-25 and OSDI-6.

The original prospective cohort study was conducted at the Clinic for Eye Diseases, University Clinical Center of Serbia, Belgrade, Serbia, from October 2024 to July 2025. The study was conducted in accordance with the Declaration of Helsinki and was approved by the Ethics Committee of the University Clinical Center of Serbia (approval No. 341/16; amendment No. 1051/23). All participants provided written informed consent before enrollment, including consent for treatment with lyophilized AM eye drops and for the use of anonymized clinical and questionnaire data for research purposes.

### 2.2. Study Population

The present secondary analysis included 40 consecutive adult patients with confirmed DED who completed both baseline and final patient-reported outcome assessments. All patients had participated in the previously reported prospective cohort and had been followed over six study visits. Because the present analysis evaluated questionnaire-based outcomes, all patient-reported data were analyzed at the patient level.

Patients were eligible for inclusion in the original cohort if they were aged 18 years or older and had a diagnosis of DED based on symptoms and objective evidence of tear film homeostasis disruption, in accordance with TFOS DEWS II-based diagnostic criteria [1,2,3]. In the original cohort, DED was defined by an OSDI score ≥ 13 and at least one objective sign of ocular surface involvement, such as TBUT < 10 s or corneal staining > 5 spots. Additional inclusion criteria included insufficient response to previous standard DED therapy, use of topical cyclosporine A for at least 3 months before enrollment, daily computer use of at least 1 h, and signed informed consent.

The exclusion criteria were ocular surgery within the previous 6 months, active ocular infection, known hypersensitivity to topical ophthalmic preparations used in the study protocol, use of topical corticosteroids within the previous month, pregnancy or lactation, immunodeficiency, and contact lens wear. No additional inclusion or exclusion criteria were applied for the present secondary analysis, except for the availability of completed NEI VFQ-25 and OSDI-6 questionnaires at both baseline and the final visit.

### 2.3. Treatment Protocol

All patients received lyophilized AM eye drops in addition to their pre-existing standard DED therapy. Background therapy, including lubricants and topical cyclosporine A, remained unchanged throughout the study period. Lyophilized AM eye drops were administered to both eyes 6 times daily, 2 drops per eye, in repeated 14-day treatment cycles. A fresh preparation was provided at each follow-up visit. The treatment and follow-up period consisted of six study visits: the baseline visit (Visit 1), followed by five follow-up visits at approximately 14-day intervals (Visits 2–6). Accordingly, the final patient-reported outcome assessment was performed at Visit 6, approximately 70 days after baseline.

Amniotic membranes were obtained after elective cesarean section following donor consent and appropriate serological screening. Tissue processing was performed under standardized conditions at the licensed Eye Bank of the University Clinical Center of Serbia. After antibiotic and antimycotic treatment, the amnion was separated from the chorion, processed into fragments, lyophilized, reconstituted with sterile water for injection, and filtered through a sterile 0.22 µm membrane filter. The final solution was stored at +5 °C, protected from light, and used within a 14-day shelf-life period. Batch traceability and basic quality control procedures, including visual inspection and pH monitoring, were maintained throughout the study.

### 2.4. Patient-Reported Outcome Measures

Patient-reported outcomes were assessed at baseline, before initiation of lyophilized AM eye drop therapy, and at the final study visit after completion of the treatment cycles. Formally validated Serbian-language versions of both the NEI VFQ-25 and OSDI-6 questionnaires were used in the present study. No study-specific translation or cultural adaptation was therefore required. The questionnaires were self-administered by the patients and completed under standardized conditions before ophthalmic examination in order to avoid any influence of clinical testing on symptom reporting.

#### 2.4.1. National Eye Institute Visual Function Questionnaire-25

Vision-related quality of life was assessed using the NEI VFQ-25, a widely used questionnaire designed to evaluate the impact of visual function on daily activities and psychosocial well-being [9]. The NEI VFQ-25 includes items covering general health, general vision, ocular pain, near activities, distance activities, social functioning, mental health, role difficulties, dependency, driving, color vision, and peripheral vision.

In the present analysis, individual NEI VFQ-25 items were evaluated at baseline and at the final visit. Particular attention was given to items related to ocular pain and discomfort, near-vision activities, distance-related visual tasks, social functioning, and dependence on others, as these domains were considered clinically relevant to DED-related symptom burden and daily visual function.

#### 2.4.2. Ocular Surface Disease Index-6

Dry eye symptoms were assessed using the OSDI-6, a shortened six-item version of the original Ocular Surface Disease Index developed to provide a concise assessment of key symptom and functional domains of ocular surface disease [10,11].

The six OSDI-6 items evaluated light sensitivity, blurred vision, difficulties with driving or night driving, difficulties watching television or similar visual tasks, discomfort in windy conditions, and discomfort in low-humidity environments. Each item was scored according to symptom frequency using the standard OSDI response structure, ranging from absence of symptoms to symptoms occurring all of the time. In the present analysis, changes in the distribution of responses for each OSDI-6 item between baseline and the final visit were evaluated.

### 2.5. Objective Ocular Surface Outcomes from the Primary Cohort

Objective ocular surface outcomes were not reanalyzed as primary endpoints in the present study, as they were the focus of the previously published primary report [19]. However, the main objective findings from the primary cohort were considered when interpreting patient-reported outcomes. These included significant prolongation of TBUT, reduction in corneal and conjunctival staining, improvement in meibomian gland quality and expressibility, and increased corneal sensitivity, with stable best-corrected visual acuity, intraocular pressure, and Schirmer I values.

The present study therefore aimed to determine whether the previously observed objective stabilization of the ocular surface was accompanied by measurable improvements in patient-perceived symptoms and vision-related quality of life.

### 2.6. Statistical Analysis

All questionnaire-based analyses were performed at the patient level. For item-level analyses, only valid paired baseline and final responses were included; non-applicable responses such as “not driving” were excluded from the denominator for the corresponding item. Descriptive statistics were used to summarize response distributions for each NEI VFQ-25 and OSDI-6 item at baseline and at the final visit. Categorical and ordinal responses are presented as frequencies and percentages.

Because questionnaire responses were ordinal and paired within the same patients, baseline-to-final changes in individual item responses were analyzed using non-parametric tests for paired ordinal data. The Wilcoxon signed-rank test was used to evaluate within-patient changes in ordinal item scores. When response-category distributions were analyzed as paired categorical data with more than two response categories, marginal homogeneity procedures were applied as appropriate.

A two-sided *p* value < 0.05 was considered statistically significant. Because this was an exploratory secondary analysis of patient-reported outcomes, *p* values were interpreted descriptively, and no formal adjustment for multiple comparisons was applied. Statistical analyses were performed using IBM SPSS Statistics, version 27.0 (IBM Corp., Armonk, NY, USA).

## 3. Results

A total of 40 patients with dry eye disease completed both baseline and final patient-reported outcome assessments and were included in this secondary analysis. All patients received lyophilized amniotic membrane eye drops in repeated 14-day treatment cycles over six study visits, while continuing their pre-existing standard dry eye therapy.

### 3.1. NEI VFQ-25 Outcomes

Changes in individual NEI VFQ-25 items between baseline and the final visit are summarized in Table 1. Global and general items, including general health, overall self-rated vision, and worry about eyesight, did not show statistically significant changes over the treatment period (*p* = 0.180, *p* = 0.102, and *p* = 0.157, respectively). These findings suggest that broad self-perceptions of health and vision remained relatively stable during follow-up.

In contrast, several symptom-related and functionally relevant NEI VFQ-25 items improved significantly. Ocular pain and discomfort, including burning, itching, or aching in and around the eyes, decreased significantly between baseline and the final visit (*p* = 0.002). At baseline, the most frequent response category was moderate ocular discomfort, whereas at the final visit responses shifted toward mild or absent discomfort.

Significant improvements were also observed in near-vision activities. The proportion of patients reporting no difficulty reading ordinary print increased from 10.0% at baseline to 25.0% at the final visit (*p* = 0.003). Similarly, the proportion of patients reporting no difficulty performing work or hobbies requiring near vision increased from 5.0% to 25.0% (*p* = 0.014). Finding objects on a crowded shelf or in a drawer also improved significantly, with the proportion of patients reporting no difficulty increasing from 22.5% to 45.0% (*p* = 0.008).

Distance-related and spatial visual tasks showed a similar pattern. Reading street signs or store names improved significantly (*p* < 0.001), with the proportion of patients reporting no difficulty increasing from 25.0% to 50.0%. Noticing objects off to the side while walking also improved significantly (*p* = 0.037), with the proportion of patients reporting no difficulty increasing from 32.5% to 55.0%. In contrast, difficulty going down steps in dim light or at night did not reach statistical significance (*p* = 0.059).

Vision-related social functioning improved in several items. Significant improvements were observed in noticing how people react to things the patient says (*p* = 0.007), going out to visit people, attending social gatherings, or going to restaurants (*p* = 0.005), and going to movies, plays, or sports events (*p* = 0.038). These changes reflected a shift toward fewer vision-related limitations in social activities.

Driving-related NEI VFQ-25 items did not show statistically significant changes, including daytime driving, night driving, and driving in difficult conditions (*p* = 0.317, *p* = 1.000, and *p* = 1.000, respectively). This may partly reflect the limited number of active drivers in the cohort and the presence of “not driving” responses.

Most items related to role limitations, frustration, loss of control, and fear of embarrassment did not change significantly. However, two dependence-related items showed statistically significant improvement. The proportion of patients reporting that they did not stay home most of the time because of their eyesight increased from 92.4% to 95.0% (*p* = 0.025). In addition, the proportion of patients reporting that they did not rely too much on what other people told them because of their eyesight increased from 89.7% to 94.0% (*p* = 0.034).

Overall, significant improvements in NEI VFQ-25 responses were observed mainly in ocular pain, near-vision tasks, selected distance and spatial tasks, social functioning, and dependence-related domains.

### 3.2. OSDI-6 Outcomes

Changes in OSDI-6 items between baseline and the final visit are summarized in Table 2. Five of the six OSDI-6 items showed statistically significant improvement.

Light sensitivity improved significantly (*p* < 0.001). At baseline, more than half of the patients reported light sensitivity often, whereas at the final visit responses shifted toward the categories “never” and “sometimes”. Blurred vision also improved significantly (*p* = 0.008), with fewer patients reporting frequent symptoms at the final visit.

Difficulty with driving or night driving showed significant improvement (*p* = 0.002), with an increase in the proportion of patients reporting no difficulty and a decrease in frequent or constant symptoms. Difficulty watching television or performing similar visual tasks also improved significantly (*p* = 0.008). Discomfort in windy conditions showed a smaller but statistically significant improvement (*p* = 0.046), with a modest shift toward less frequent symptoms.

Discomfort in low-humidity environments did not reach statistical significance (*p* = 0.059), although the response distribution showed a trend toward less frequent symptoms. This item appeared to be less responsive to treatment than the other OSDI-6 symptom domains.

## 4. Discussion

In this secondary analysis of a prospective cohort of patients with DED treated with lyophilized AM eye drops, we observed significant improvements in several patient-reported domains of vision-related quality of life and dry eye symptoms. The most pronounced changes were found in ocular pain and discomfort, near-vision activities, selected distance-related and spatial visual tasks, social functioning, and dependence-related items of the NEI VFQ-25. In parallel, five of the six OSDI-6 items improved significantly, including light sensitivity, blurred vision, difficulty with driving or night driving, difficulty watching television, and discomfort in windy conditions. These findings suggest that the previously reported objective stabilization of the ocular surface in this cohort was accompanied by measurable improvements in patient-perceived symptom burden and daily visual functioning [19]. However, the magnitude of these changes was not uniform across domains; greater improvements were observed in ocular discomfort and visually demanding activities than in the dependence-related items, where the absolute changes were relatively small.

A key finding of the present analysis is that improvements were not distributed uniformly across all questionnaire domains. General items, such as overall health, self-rated vision, and worry about eyesight, did not change significantly. This is clinically plausible, as these broad constructs may be influenced by age, systemic comorbidities, chronicity of disease, individual expectations, and general health perception rather than by short- to medium-term changes in ocular surface status alone. In contrast, items more directly related to ocular surface discomfort and visually demanding daily tasks showed significant improvement. This pattern supports the internal consistency of the findings, as improvements were observed predominantly in domains likely to be affected by tear film instability, epithelial irregularity, and ocular surface discomfort.

The improvement in ocular pain and discomfort is particularly relevant. Patients with DED commonly experience burning, stinging, foreign-body sensation, itching, and fluctuating discomfort, symptoms that may persist despite conventional therapy [1,2,7,8]. In the present study, the significant improvement in the NEI VFQ-25 ocular pain item was accompanied by improvement in several OSDI-6 symptom domains, including light sensitivity and blurred vision. These changes are consistent with the concept that stabilization of the tear film and improvement of epithelial surface integrity may reduce sensory irritation and visual fluctuation. Importantly, these patient-reported improvements complement the previously reported objective findings in the same cohort, including significant TBUT prolongation, reduced corneal and conjunctival staining, improved meibomian gland quality and expressibility, and increased corneal sensitivity [19].

Significant improvements were also observed in near-vision activities. Reading ordinary print, performing work or hobbies requiring near vision, and finding objects on a crowded shelf or in a drawer all improved between baseline and the final visit. These activities require a stable optical surface and are highly sensitive to tear film instability. Even mild irregularity of the tear film can induce fluctuating vision, visual fatigue, and reduced reading comfort [5,6]. Therefore, the improvement in these NEI VFQ-25 items is consistent with the previously observed increase in TBUT and reduction in ocular surface staining. In practical terms, these findings indicate that the observed ocular surface stabilization was accompanied by improved performance of everyday near-vision tasks, which are especially relevant in patients who use computers or perform visually demanding work.

Distance-related and spatial visual tasks also improved, particularly reading street signs and noticing objects off to the side while walking. Although DED is not traditionally considered a disease that primarily impairs distance vision, tear film instability can degrade optical quality and cause intermittent blur, glare, and visual fluctuation. The observed improvement in these items may therefore reflect a reduction in tear film-related optical disturbance rather than a change in best-corrected visual acuity, which remained stable in the primary analysis [19]. This distinction is important: standard visual acuity may remain unchanged even when patients report improvements in functional vision during real-life visual tasks.

The improvement in vision-related social functioning adds another patient-centered dimension to the findings. Significant changes were observed in items related to noticing other people’s reactions, going out to visit people or attend social gatherings, and participating in activities such as movies, theater, or sports events. These domains are not direct measures of ocular surface biology, but they capture the broader functional consequences of symptomatic DED. Persistent discomfort, photophobia, blurred vision, and visual fatigue may reduce confidence in social environments and limit participation in daily activities. The improvement in these items indicates that changes in ocular symptoms were accompanied by improvements in broader aspects of social functioning and quality of life.

The OSDI-6 results further support the consistency of the observed patient-reported improvements. Five of the six items showed statistically significant improvement, with the strongest changes observed for light sensitivity, blurred vision, and difficulty with driving or night driving. These items represent core OSDI/OSDI-6 symptom domains [10,11] and are clinically consistent with tear film instability and ocular surface irregularity, which are central features of DED [1,2,3]. In contrast, discomfort in low-humidity environments showed only a trend toward improvement and did not reach statistical significance. This may indicate that environmental stressors such as dry air remain difficult to modify, particularly in patients with chronic DED, meibomian gland dysfunction, prolonged screen exposure, air conditioning, or heating. This finding is clinically useful because it suggests that even when ocular surface stability improves, some environmental triggers may continue to require additional protective strategies.

The apparently different findings for driving-related outcomes should be interpreted in the context of the distinct constructs assessed by the two questionnaires. The NEI VFQ-25 driving items assess broader vision-related difficulty with daytime driving, night driving, and driving under difficult conditions, whereas the OSDI-6 item focuses more specifically on the frequency of ocular-surface-related difficulty during driving or night driving. In addition, the number of evaluable responses for the NEI VFQ-25 driving items was reduced because some participants reported that they did not drive. Therefore, the absence of significant change in the NEI VFQ-25 driving items and the improvement observed in the OSDI-6 driving/night-driving item should not necessarily be regarded as internally inconsistent.

The present findings also contribute to the broader discussion regarding the relationship between signs and symptoms in DED. A well-recognized feature of DED is the imperfect correlation between objective ocular surface signs and subjective symptoms. Some patients report severe symptoms despite modest clinical findings, whereas others have marked staining or tear film abnormalities with relatively few complaints [7,8]. In this cohort, however, the direction of change was coherent: objective improvements reported previously were accompanied by improvement in multiple patient-reported outcomes. This does not prove a direct causal relationship, but it indicates that the observed clinical stabilization was accompanied by improvements in multiple patient-reported outcomes.

From a biological standpoint, the pattern of patient-reported improvement is compatible with the proposed mechanism of AM-derived therapies. Amniotic membrane contains extracellular matrix components, growth factors, anti-inflammatory mediators, and molecules involved in epithelial repair and immune modulation [13,14,15,16,17]. These properties may support restoration of epithelial barrier integrity, reduction in inflammatory activation, and improvement of the ocular surface microenvironment. In the primary analysis of this cohort, lyophilized AM eye drops appeared to improve tear film quality and ocular surface integrity rather than aqueous tear production, as TBUT, staining, meibomian gland parameters, and corneal sensitivity improved, whereas Schirmer I values did not change significantly [19]. The present patient-reported results are consistent with this interpretation: symptoms related to visual fluctuation, discomfort, light sensitivity, and functional vision improved, while broader general health-related perceptions remained stable.

The potential neurotrophic dimension of AM-derived therapy may also be relevant. Increased corneal sensitivity was previously observed in this cohort [19], and this may reflect improved epithelial–neural interaction or a more stable ocular surface environment. Although the present study did not directly measure corneal nerve morphology or tear biomarkers, improved ocular comfort and reduced symptom frequency are compatible with changes that may extend beyond lubrication alone. This remains a hypothesis and should be confirmed in future studies using objective biomarkers, tear proteomics, in vivo confocal microscopy, or other methods capable of characterizing epithelial, inflammatory, and neurosensory changes.

An important strength of this study is its patient-centered focus. While the primary report demonstrated objective clinical improvement, the present analysis addresses whether those changes were reflected in outcomes that matter directly to patients. The use of both NEI VFQ-25 and OSDI-6 allowed assessment of complementary aspects of disease burden. NEI VFQ-25 captured broader functional and psychosocial consequences of visual symptoms, whereas OSDI-6 provided a concise measure of key dry eye symptom domains [9,10,11]. This combined approach provides a more complete picture of the observed clinical and patient-reported changes than objective clinical testing alone.

Several limitations should be acknowledged. First, this was a secondary analysis of a single-arm prospective cohort without a randomized control group. Therefore, the specific contribution of lyophilized AM eye drops cannot be fully separated from placebo effects, regression to the mean, natural symptom fluctuation, or the effect of continued background therapy. However, all patients had persistent symptoms despite standard therapy before enrollment, and concomitant treatment was kept unchanged during follow-up, supporting the interpretation of lyophilized AM eye drops as an adjunctive intervention. Second, the sample size was modest and the study was conducted at a single tertiary referral center, which may limit generalizability to broader or milder DED populations. Third, the analysis focused on item-level questionnaire responses rather than composite NEI VFQ-25 domain scores, total OSDI-6 scores, or minimal clinically important differences. Fourth, multiple questionnaire items were tested, and the *p* values should therefore be interpreted descriptively and cautiously. Finally, the study did not include direct biomarker measurements, so mechanistic explanations related to extracellular matrix restoration, inflammatory modulation, proteolytic burden, or neurotrophic effects remain biologically plausible interpretations based on previous literature rather than direct findings of the present analysis.

Future research should include randomized controlled trials comparing lyophilized AM eye drops with standard therapy, placebo, or other biologically based ocular surface treatments. Longer follow-up is needed to determine the durability of patient-reported improvements after treatment discontinuation. Future studies should also calculate composite NEI VFQ-25 and OSDI-6 scores, evaluate minimal clinically important differences, and integrate objective biomarkers such as tear cytokines, matrix metalloproteinase activity, tear film lipid-layer parameters, and corneal nerve imaging. Such studies would help identify which DED phenotypes are most likely to benefit from AM-derived therapy and whether improvement in patient-reported outcomes can be predicted by baseline ocular surface or biomarker profiles.

Overall, this secondary analysis shows that treatment with lyophilized AM eye drops was associated not only with previously reported objective ocular surface improvements, but also with improvements in multiple patient-reported outcomes. The observed ocular surface improvements were accompanied by improvements in ocular discomfort, daily visual function, social participation, and key dry eye symptoms. These findings support further controlled investigation of lyophilized AM eye drops as a potential biologically based adjunctive approach in multimodal DED management, particularly in patients with persistent symptoms and tear film instability despite standard therapy.

## 5. Conclusions

In this secondary analysis of a prospective cohort of patients with dry eye disease, treatment with lyophilized amniotic membrane eye drops was associated with significant improvements in several patient-reported outcomes, including ocular pain and discomfort, near-vision activities, selected distance-related visual tasks, social functioning, dependence-related items, and key dry eye symptoms assessed by OSDI-6.

These findings complement the previously reported objective improvements in tear film stability, ocular surface staining, meibomian gland function, and corneal sensitivity observed in the same cohort. Together, the objective and patient-reported results suggest that treatment with lyophilized amniotic membrane eye drops was associated with improvements extending beyond ocular surface signs to daily visual function and symptom burden.

Although the single-arm design and exploratory nature of this secondary analysis limit causal interpretation, the observed pattern of improvement supports further controlled investigation of lyophilized amniotic membrane eye drops as a potential biologically based adjunctive approach in dry eye disease management. Further randomized controlled studies with longer follow-up, composite questionnaire scores, minimal clinically important difference analysis, and biomarker-based phenotyping are warranted to confirm these findings and identify patients most likely to benefit from this treatment approach.

## Figures and Tables

**Table 1:** NEI VFQ-25 items showing statistically significant improvement between baseline and the final visit.

Item	Domain	Description	*p* Value	Direction of Change	Item
4	Ocular pain	Pain or discomfort in or around the eyes	0.002	Less pain/discomfort; more mild or absent symptoms	4
5	Near activities	Reading ordinary print	0.003	More patients without difficulty	5
6	Near activities	Work or hobbies requiring near vision	0.014	More patients without difficulty	6
7	Near activities	Finding objects on a crowded shelf or in a drawer	0.008	More patients without difficulty	7
8	Distance activities	Reading street signs or store names	<0.001	More patients without difficulty	8
10	Peripheral vision	Noticing objects off to the side while walking	0.037	More patients without difficulty	10
11	Social functioning	Noticing how people react to things said	0.007	Fewer vision-related social limitations	11
13	Social functioning	Visiting people, attending gatherings, or going to restaurants	0.005	Fewer vision-related social limitations	13
14	Social functioning	Going to movies, plays, or sports events	0.038	Fewer vision-related social limitations	14
20	Dependency	Staying home most of the time because of eyesight	0.025	Fewer dependence-related limitations	20
23	Dependency	Relying on what other people say because of eyesight	0.034	Less reliance on others	23

**Table 2:** OSDI-6 item-level changes between baseline and the final visit.

Item	Symptom Domain	*p* Value	Direction of Change
1	Light sensitivity	<0.001	Shift toward “never” or “sometimes”
2	Blurred vision	0.008	Shift toward “never” or “sometimes”
3	Difficulty with driving or night driving	0.002	Shift toward less frequent symptoms
4	Difficulty watching television or similar visual tasks	0.008	Shift toward less frequent symptoms
5	Discomfort in windy conditions	0.046	Mild shift toward less frequent symptoms
6	Discomfort in low-humidity environments	0.059	Trend toward improvement; not statistically significant

## Data Availability

The original contributions presented in this study are included in the article. Further inquiries can be directed to the corresponding authors.
